# Physical Condition and Activity of Daily Living among HIV Patients Infected through Blood Products in Japan

**DOI:** 10.2188/jea.12.383

**Published:** 2007-11-30

**Authors:** Takuhiro Yamaguchi, Shuji Hashimoto, Shin-ichi Oka, Kazuyuki Yoshizaki, Satoshi Kimura, Katsuyuki Fukutake, Takuma Shirasaka

**Affiliations:** 1 Department of Biostatistics / Epidemiology and Preventive Health Sciences, School of Health Sciences and Nursing, University of Tokyo.; 2 Department of Hygiene, Fujita Health University School of Medicine.; 3 AIDS Clinical Center, International Medical Center.; 4 Department of Medical Science I, School of Health and Sport Sciences, Osaka University.; 5 Department of Infectious Disease, School of Medicine, University of Tokyo.; 6 Department of Laboratory Medicine, Tokyo Medical College Hospital.; 7 Osaka National Hospital

**Keywords:** HIV infection, blood products, CD4 cell counts, physical condition, activity of daily living (ADL), antiretroviral treatments

## Abstract

Objective: To examine the present status and trends in physical condition and activity of daily living (ADL) among patients infected HIV by blood products in Japan.

Methods: Data from a survey of 605 HIV patients infected through blood products were available quarterly between April 1997 and March 2000. Physical condition (summary index of 13 symptoms) and ADL in the first quarter of 2000 were assessed by comparing proportions of patients in good physical condition and having good ADL according to the level of CD4 counts and the use of antiretroviral treatments. Trends in those proportions during the study period were investigated, and these trends were also assessed by the changes in CD4 counts and antiretroviral treatments.

Results and Conclusions: The proportion of patients in good physical condition was 70.6% and having good ADL was 65.7% in the first quarter of 2000, which was associated with the CD4 counts and antiretroviral therapy. The proportion of patients in good physical condition decreased from 79.2% to 66.2%, and ADL also decreased from 72.1% to 61.3% during the study period. These declines were presumably associated with the changes in CD4 counts and antiretroviral treatments.

In Japan, according to the report of HIV/AIDS surveillance by the Ministry of Health, Labor and Welfare, the numbers of HIV patients infected through the use of blood coagulation factor products (blood products) was 1,432, accounting for 27% of all HIV-infected patients in Japan.^1^^,^^2^ Among the patients, 642 developed AIDS and 493 died. It is a challenge for both public health and medicine to grasp the clinical status of these HIV-infected patients and to prevent them from developing AIDS in Japan.

In recent years, it was shown that the use of highly active antiretroviral therapy, including combination regimens such as two nucleoside reverse transcriptase inhibitors plus one protease inhibitor, had significant impact on the CD4 cell counts and HIV-RNA levels,^3^^-^^6^ and resulted in a decline in mortality and morbidity.^7^ Nevertheless, some treatments could lead to adverse effects^8^^,^^9^ and the problem of adherence,^10^^-^^12^ and there is a need to collect data on the quality of life (QOL) in HIV-infected patients.^13^^,^^14^ Assessment of health-related QOL has been included in clinical trials,^15^ as well as in major health policy studies and cohort studies.^16^ Such assessment of health status and QOL for populations of patients with HIV infection is useful and should be done more frequently.^17^ The goal is to prolong life, minimize symptoms, and optimize patient QOL.^8^^,^^14^

In Japan, a survey and research program for patients with HIV infection through blood products has been carried out since fiscal year of 1993 with the support of the Ministry of Health, Labor and Welfare. This program is intended to help HIV carriers infected through the use of contaminated blood products and suffering from reduced immune functions, to prevent them from developing HIV-related symptoms in daily living by providing health management expenses and requesting them to report their health status. When HIV-infected patients through blood products participate in this program, they need to make an application for it and to be recognized as the subject of it. Therefore, all the HIV-infected patients by blood products in Japan are not the subjects of the program. Also, if the subjects develop AIDS, they will be excluded from this survey. Details of the survey and research program are available.^18^^,^^19^ Recently, we collected data from the above-mentioned survey of HIV patients infected through blood products and investigated trends in CD4 cell counts, plasma HIV-RNA levels and the use of antiretroviral treatments.^19^ It was suggested that improved CD4 counts and HIV-RNA levels were attributable to the introduction and widespread adoption of new and effective combination regimens in the population under study. On the other hand, in this survey, data on thirteen clinical symptoms and activity of daily living (ADL) were self-reported on a quarterly basis. Symptoms are the most specific patient-reported measures of health status,^17^ and have a direct and significant impact on QOL.^13^^,^^20^ Physical functioning and ADL are also associated with health-related QOL.^17^^,^^21^ There are a few cohort or longitudinal studies that have examined the trend in health-related QOL outcomes and those relationships with changes in surrogate markers such as CD4 counts and especially with treatment history.^22^ Hence, we think it will be valuable for physicians and other health care providers if we were to investigate the present status and trends of such outcomes in this population, as well as the associations with CD4 counts and antiretroviral treatments and changes in those variables.

The purpose of the present study is to examine the current status and trends in physical condition and ADL among HIV patients infected through blood products in Japan. We also examine the relationships with CD4 cell counts, antiretroviral treatment and changes in these variables.

## MATERIALS AND METHODS

Data from the above-mentioned survey of HIV patients infected through blood products in Japan were available. Our study subjects consisted of 605 patients who were under observation on April 1, 1997. The characteristics of the subjects are shown in Table 1. About 96% of them were men. We used the data on their clinical status (CD4 cell counts, antiretroviral treatments) reported from their treating physicians as well as on their health status (physical condition and ADL) reported by the patients themselves on a quarterly basis between April 1997 and March 2000. All patients gave their written informed consent.

**Table 1.** Characteristics of the study subjects.

		Number	(%)
Total number of the study subjects		605	(100.0)

Age in the second quarter of 1997	-9 years	0	(0.0)
	10-19 years	76	(13.0)
	20-29 years	261	(44.6)
	30-39 years	163	(27.9)
	40-49 years	62	(10.6)
	50-59 years	15	(2.6)
	60- years	8	(1.4)
	unknown	20	
	mean age; 30 years (range 11-72 years)		

Disease stage in the first quarter of 2000	infected with HIV	567	(93.7)
	developed AIDS or death*	36	(6.0)
	unknown**	2	(0.3)

* excluded from the survey from the time they developed AIDS

** drop-out from the survey during the study period

The patients’ physical condition and ADL were self-reported using a questionnaire developed especially for this survey. Physical condition here includes 13 symptoms, which are anorexia, dysgeusia, dry mouth, stomatitis, diarrhea, weight loss, shortness of breath, fatigue, numbness or tingling in arms or legs, fever, eczema or itching, nanowing of visual field, and insomnia. They were asked whether or not they had experienced these symptoms. These items are answered on a scale of 5 response categories, with 1 for “not at all,” 3 for “somewhat” and 5 for “very much,” and scored with a lower score indicating a positive condition. The values of Cronbach’s alpha of scores of the thirteen items ranged 0.90 to 0.91 for the quarters during our study period, indicating that these items showed high internal consistency. Hence we used the simply averaged score of the above-mentioned 13 items as an overall measure of physical condition. Patients whose score was less than 3 were considered to be “in good physical condition.” The non-weighted and weighted kappa values of this summary score for 451 patients who reported it and did not change treatment regimens between the second and third quarter of 1997 were 0.80 and 0.84, respectively. The high reproducibility of this summary score was also shown. The item for ADL is on a scale of 8 response categories, with 1 for “I have no limitations in daily activities,” 2 for “I have some disability but have no difficulties in performing daily activities,” 3 for “I need some effort to perform daily activities,” 4 for “I can only perform minimum daily activities,” 5 for “I sometimes need someone’s help in daily activities,” 6 for “I need someone’s help in daily activities, and sometimes need medical care,” 7 for “I cannot move by myself. I need medical care, nursing, and help at home,” and 8 for “I am on bed all day. I need to be hospitalized to receive medical care and nursing.” Thus, the lower score indicates positive activity. Patients whose score was 1 or 2 were treated as “having good ADL.” The non-weighted and weighted kappa values of this score for 420 patients who reported it and did not change treatment regimens between the second and third quarter of 1997 were 0.87 and 0.91, respectively, showing high test-retest reliability.

The present physical condition and ADL in the first quarter of 2000 were assessed according to the level of CD4 cell counts and the use of antiretroviral treatments. We examined the proportion of patients who were in good condition, that is, the proportion of patients whose summary score of the 13 symptoms were less than 3, and the proportion of patients with good ADL, that is, the proportion of patients whose ADL score was 1 or 2. The CD4 lymphocyte counts were classified into four categories (less than 200 /*μ*l, between 200 and 349 /*μ*l, between 350 and 499 /*μ*l, and 500+ /*μ*l). Also, treatments of antiretroviral agents were classified into five categories: given no treatment (No treatment), treatment including only one nucleoside reverse transcriptase inhibitor (RTI 1), including only two RTIs (RTI 2), including at least two RTIs and one protease inhibitor (RTI 2+PI 1), and other treatments (Other). In this analysis, we did not use the data of patients who developed AIDS or died during our study period.

We also examined trends in the proportions of patients who were in good condition and with good ADL from the second quarter of 1997 to the first quarter of 2000. In our population, 36 patients developed AIDS or died through the first quarter of 2000. Although they were excluded from the survey and their data were not available from the quarter they progressed to AIDS or died, we did not exclude these patients from our analyses and treated them as having the poorest physical condition, ADL and CD4 cell counts, from that quarter. Thus, in our trend analyses, we counted them among patients who were not in good physical condition and did not have good ADL, and we assigned them a value of 0 for CD4 cell counts. Especially for investigating the trend in CD4 cell counts, we used the median value in the population as an endpoint. The reason for such handling was because if we excluded such patients from the analyses, we might overestimate such measures.

To investigate associations of those trends in physical condition and ADL with changes in the CD4 counts and antiretroviral treatments, we examined the trends in CD4 counts and proportions of patients in good condition and with good ADL according to the history of antiretroviral treatments in the second quarter of each year during our study period. Because of the limited sample size, we selected and classified treatment histories as six groups with “No treatment → No treatment → No treatment,” “No treatment → RTI 2+PI 1 → RTI 2+PI 1,” “RTI 2 → RTI 2 → RTI 2,” “RTI 2 → RTI 2 → RTI 2+PI 1,” “RTI 2 → RTI 2+PI 1 → RTI 2+PI 1,” and “RTI 2+PI 1 → RTI 2+PI 1 → RTI 2+PI 1.” For example, “No treatment → RTI 2 → RTI 2+PI 1” means that the corresponding patients received no treatments in the second quarter of 1997, two RTIs in the second quarter of 1998, and at least two RTIs and one PI in the second quarter of 1999.

We obeyed several terms of agreement about secret information on this survey and did not utilize information such as name or address that could identify any patients.

## RESULTS

**Figure 1.**
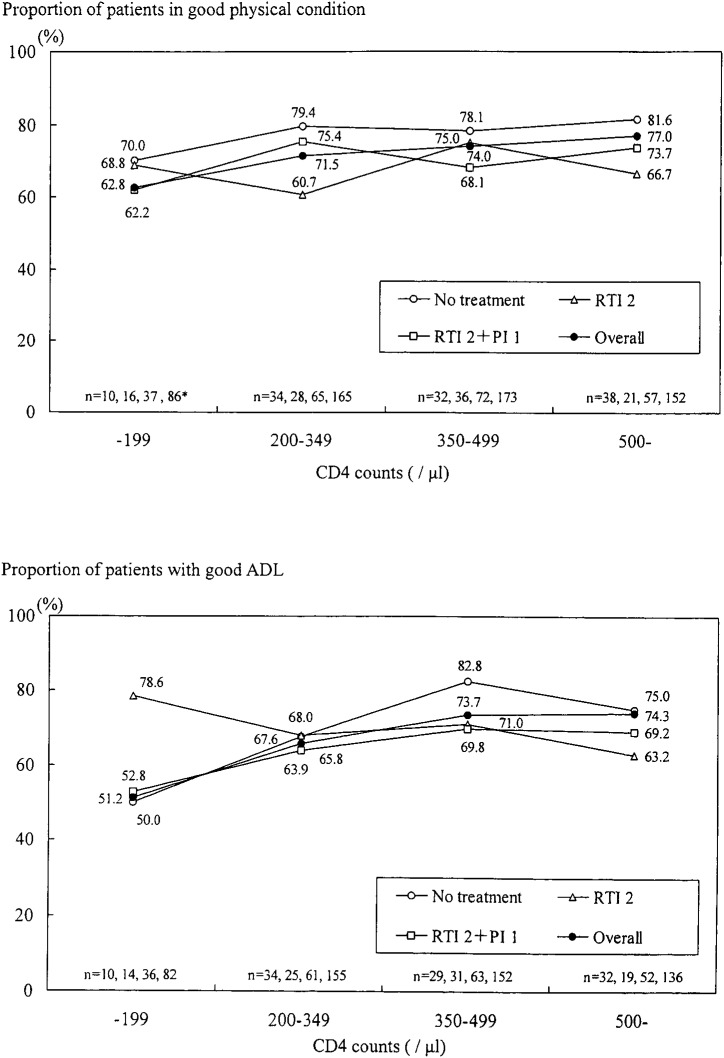
Present physical condition (top) and activity of daily living (ADL, bottom) according to CD4 cell counts and antiretroviral treatments in the first quarter of 2000. *Sample size in order, “No-treatment”, “RTI 2”, “RTI 2+PI 1” and “Overall”

The mean (median) value of CD4 counts was 395 (375)/*μ*l, ranging from 2 to 1278/*μ*l in the first quarter of 2000. Proportions of patients whose CD4 counts were less than 200/*μ*l was 79/503 (15.7%), 145/503 (28.8%) with 200-349/*μ*l, 153/503 (30.4%) with 350-499/*μ*l and 126/503 (25.0%) with 500+/*μ*l. The proportions of patients who were in good physical condition and showed good ADL were 386/547 (70.6%) and 326/496 (65.7%), respectively. Figure 1 shows the distribution of those proportions according to the CD4 counts and the classification of antiretroviral treatments (illustrated for “No treatment,” “RTI 2,” and “RTI 2+PI 1” groups because of limited sample size, as well as for “Overall” patients in our population) in the first quarter of 2000. Both for physical condition and ADL, those proportions became larger as CD4 counts increased.

As for physical condition, patients who received no treatment were in better condition than those who received other treatments such as “RTI2” or “RTI 2+PI 1.” This relation did not change according to the level of CD4 counts. On the other hand, for ADL, the proportions in the “No treatment” group were not different from those of other groups with low CD4 counts (less than 350 /*μ*l). For patients whose CD4 counts were equal or more than 350 /*μ*l, patients in the “No treatment” group exhibited better ADL than other groups.

**Figure 2.**
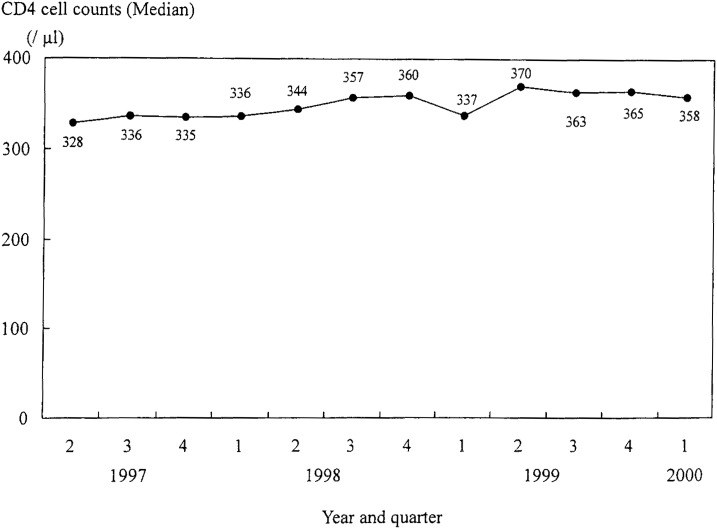
Trend in the median value of CD4 cell counts from the second quarter of 1997 to the first quarter of 2000.

Figure 2 shows the trend in CD4 median counts from the second quarter of 1997 to the first quarter of 2000. The median value of CD4 counts increased from 328 to 358 during the study period.

**Figure 3.**
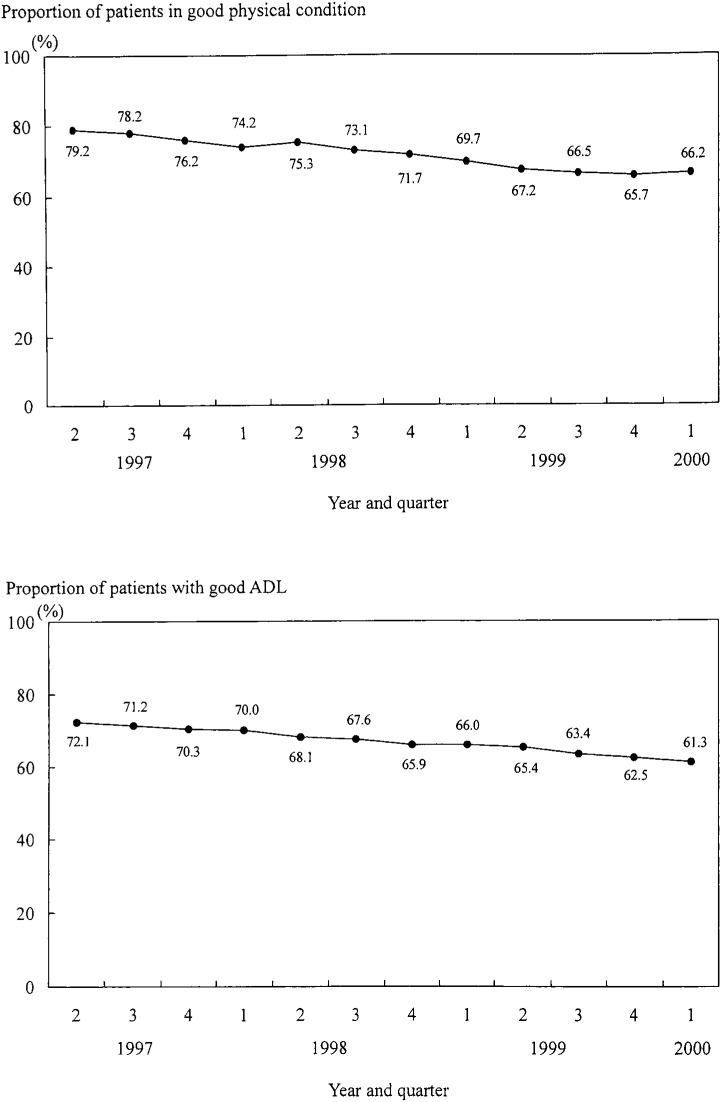
Trend in the proportions of patients in good physical condition (top) and with good activity of daily living (ADL, bottom) from the second quarter of 1997 to the first quarter of 2000.

For physical condition, the proportion of patients in good condition was 79.2% in the second quarter of 1997, but there was about a 10% reduction in the proportion in the first quarter of 2000 (66.2%). This was also the case for ADL; the proportion of patients with good ADL changed from 72.1% to 61.3% during the same period. Both physical condition and ADL declined in this population (Figure 3).

**Figure 4.**
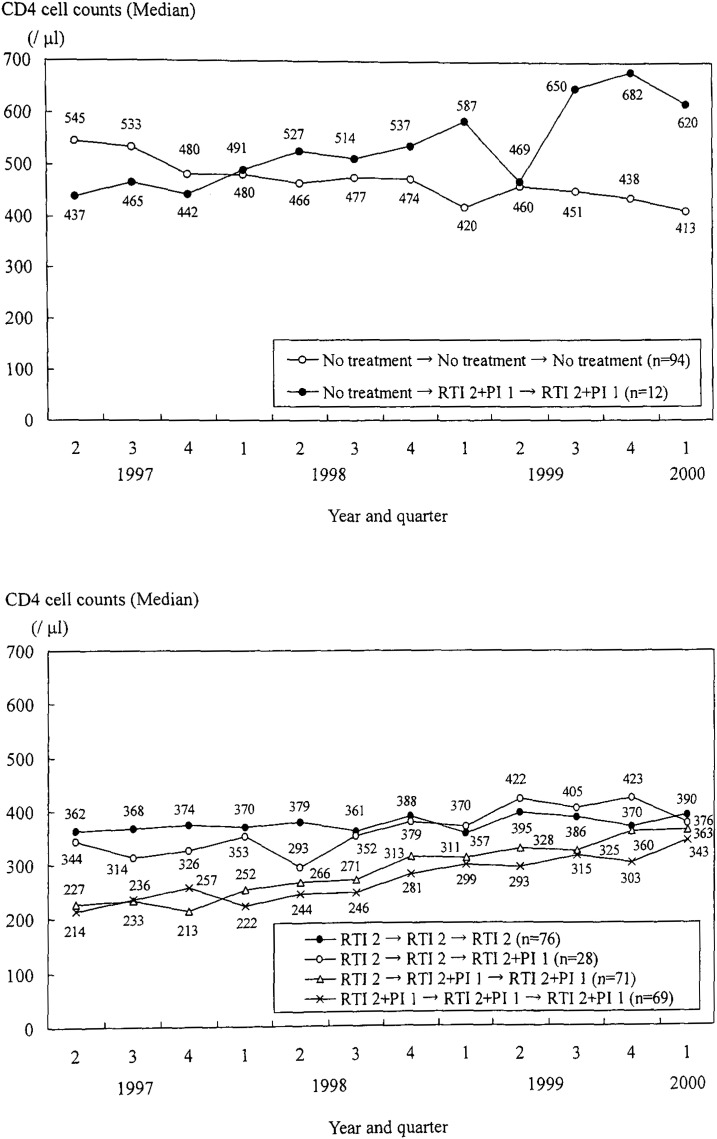
Trend in the median value of CD4 cell counts according to the changes in antiretroviral treatments from the second quarter of 1997 to the first quarter of 2000.

Figure 4 shows the trend in CD4 median counts according to the classification of antiretroviral treatments in the second quarter of each year (that is, according to the groups of antiretroviral treatment histories). For patients whose treatment history was “No treatment → No treatment → No treatment,” CD4 counts decreased from 545 to 413/*μ*l but consistently remained above 350/*μ*l. For “RTI 2 → RTI 2 → RTI 2” patients, the CD4 count was steady during the study period. For patients in other groups of antiretroviral treatment histories, the CD4 median counts increased, especially after changing to “RTI 2+PI 1” treatment. For patients in groups “RTI 2 → RTI 2+PI 1 → RTI 2+PI 1” or “RTI 2+PI 1 → RTI 2+PI 1 → RTI 2+PI 1,” CD4 counts increased from about 200 to about 350/*μ*l during the study period.

**Figure 5.**
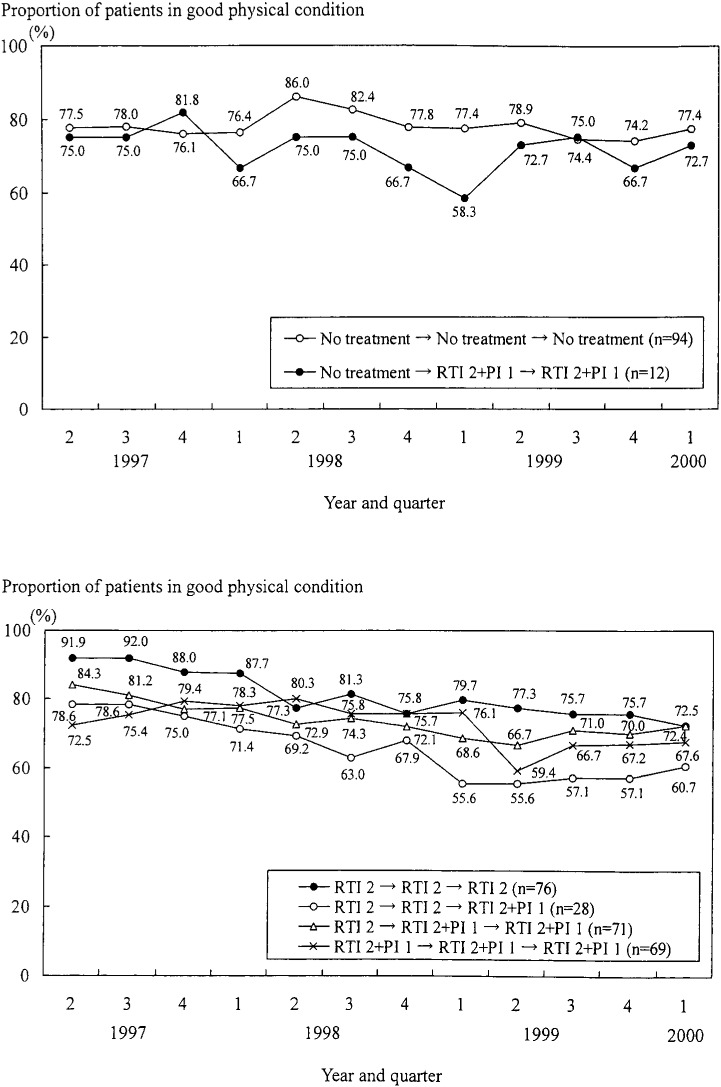
Trend in the proportion of patients in good physical condition according to the changes in antiretroviral treatments from the second quarter of 1997 to the first quarter of 2000.

On the other hand, with regard to the relationship between the trend in physical condition and changes in antiretroviral treatments, the proportion of patients in good condition decreased about 10 to 20% in almost all the groups of treatment histories except for “No treatment → No treatment → No treatment” group (Figure 5). The trend in this group was steady at around 80%. This relationship between the trend in proportion of patients in good physical condition and changes in antiretroviral treatment was different from the relationship between the trend in CD4 counts and the treatment history.

**Figure 6.**
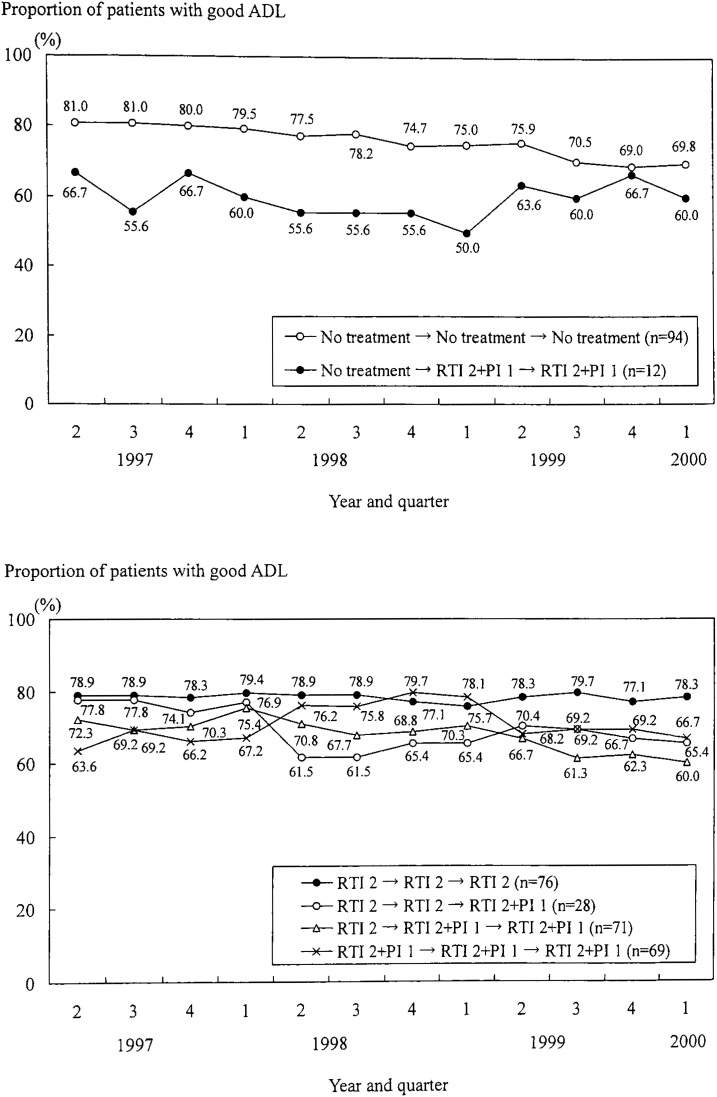
Trend in the proportion of patients with good activity of daily living (ADL) according to the changes in antiretroviral treatments from the second quarter of 1997 to the first quarter of 2000

In contrast to physical condition, there were downward trends in ADL among almost all the groups of treatment history (Figure 6). The proportion of patients showing good ADL in the “No treatment → No treatment → No treatment” group decreased from 81.0% in the second quarter of 1997 to 69.8% in the first quarter of 2000. In the group “RTI 2 → RTI 2 → RTI 2,” the proportion of patients with good ADL remained around 78 to 80% during the study period.

## DISCUSSION

With the development of new antiretroviral agents to slow disease progression, patients are living longer after diagnosis and initial treatment. Hence interest has increasingly focused on the quality of patient survival.^23^ We assessed the physical condition and ADL in HIV-infected patients, but our study had several limitations. First, HIV-infected patients who progressed to AIDS were not subjects of our study. Although in trend analyses, we did not exclude these patients and treated them as having the poorest physical condition, ADL and CD4 cell counts, these handling of data may have affected the results. Second, we could not use data on all the HIV-infected patients through blood products in Japan (about 80%). In addition, study variables were measured at an unspecified time within a quarter. Measurement of ADL may have been limited by reliance on only one scale. The time from HIV infection was not measured in this survey and we could not consider the effect of it in the analyses. Besides, we could not take account of the effect of age. Although there exists these limitations, it is important to examine the present status and trends in physical condition and ADL among HIV-infected patients through blood products so as to help to form health policy and improve the lives of patients with HIV. These issues merit the attention of clinicians treating them.

Analyses of the present status showed that about 70% of patients in our population were in good condition, showed good ADL, and their physical condition and ADL were well controlled. The outcomes were related to the level of CD4 counts. Lower CD4 cell counts were associated with poorer physical condition and lower ADL. These findings are consistent with the results of previous studies.^17^^,^^20^^,^^21^^,^^24^^,^^25^ Antiretroviral treatments were also related to physical condition and ADL, but the relationships were different. Patients who received some treatments were in poorer physical condition than patients not receiving treatments. This relationship between physical condition and treatments did not change with the CD4 count. For ADL, patients whose CD4 count was 350+/*μ*l and who received some antiretroviral treatments were in poorer condition and showed lower ADL than patients who did not receive treatments. When the CD4 count was less than 350/*μ*l, however, ADL for patients that did not receive treatments was not different from those of other patients receiving some treatments. This suggested that ADL was mainly related to CD4 counts, especially for patients with low CD4 counts.

Because the period when CD4 counts increased was almost the same as the time treatment was changed to “RTI 2+PI 1” and these combination therapies were found to increase CD4 cell counts, it was suggested the improvement of CD4 cell counts was clearly linked to the changes in antiretroviral treatment in this population. Contrary to the case for CD4 cell counts, the proportion of patients in good physical condition decreased except for those with no previous antiretroviral treatments. The physical condition consisted of 13 symptoms that primarily reflected the side effects of antiretroviral treatment. Hence we think this decline was mainly due to the adverse effects of antiretroviral treatments in our population.

The proportion of patients with good ADL also decreased in almost all groups of antiretroviral treatment histories. In the “No treatment → No treatment → No treatment” group, this decline was very similar to the change in CD4 cell counts. Thus, as was also the case in the “RTI 2 → RTI 2 → RTI 2” group, the proportion was steady while CD4 counts did not change during the study period. The CD4 count is a surrogate marker of disease progression, and it was suggested that the trend in ADL was mainly related to the change in these counts. Other study showed that changes in CD4 counts were significantly related to improvements in the Physical Health Summary Score.^25^ Because CD4 counts decreased for patients not receiving antiretroviral treatments, the proportion of patients showing good ADL was diminished in that group. In the “RTI 2 → RTI 2 → RTI 2” group, the CD4 counts remained steady so that the proportion was also unchanged. On the other hand, patients in other groups of treatment histories were not so high level although CD4 counts increased (that is, their CD4 median counts were less than 350/*μ*l in the second quarter of 1997 and at most 350 to 400/*μ*l during the study period). Combining the result discussed above that the present ADL was mainly related to CD4 counts, we inferred that the proportions in those groups were decreased mainly because of the low CD4 level and consequently there was a downward trend in the proportion of patients with good ADL in our population. Another study showed that patients displaying no change or increase of CD4 counts declined in disability.^23^

In conclusion, about 70% of patients in this population were in good physical condition and had good ADL in the first quarter of 2000. Some patients whose CD4 cell count level was low and/or who received combination therapy were in poor physical condition and showed low ADL. We must improve their physical condition and ADL by considering not only the clinical benefit of antiretroviral therapy (improvement of CD4 counts) but also side effects or other problems of antiretroviral treatments. Physical condition and ADL worsened from the second quarter of 1997 to the first quarter of 2000 in our population. It was suggested that this deterioration in physical condition was primarily due to changes in the antiretroviral treatments, while that of ADL mainly related to the changes in CD4 cell counts. Antiretroviral treatments continue to evolve, and regimens will become more complex.^26^ More complicated assessments of changes of those treatments may be needed to examine physical condition and ADL.
